# History of Dentistry

**Published:** 1878-04

**Authors:** 


### HISTORY OF DENTISTRY.

We have had some inquiry recently in reference to the publication of "The History of Dentistry," which has been in course of preparation for two or three years, with which the editor of the REGISTER has been somewhat connected.

In reply to such enquiries, suffice it to say, the work is progressing, but not as rapidly as was at one time proposed, and this for two or three reasons. During the last two years and a half, a large amount of additional labor in another direction has devolved upon the editor. Again, the times in a financial aspect have not been propitious for the issue of such works.

And again, a work presenting somewhat the history of dentistry was issued about two years ago, and it may be well that it become digested before anything more of the same sort is put forth. And still again, the labor and research requisite for the proper preparation of such a work is far greater than was at first anticipated. We trust the work will appear at the time and in such form as will at least answer a modest expectation.

